# Termite Nests as an Abundant Source of Cultivable Actinobacteria for Biotechnological Purposes

**DOI:** 10.1264/jsme2.ME13183

**Published:** 2014-06-06

**Authors:** Nikhom Sujada, Rungroch Sungthong, Saisamorn Lumyong

**Affiliations:** 1 Department of Biology, Faculty of Science, Chiang Mai University, Chiang Mai 50200, Thailand; 2 Instituto de Recursos Naturales y Agrobiología de Sevilla (IRNAS-CSIC), Seville 41080, Spain

**Keywords:** termite nest, Actinobacteria, functional diversity, 16S rRNA gene, bioactive compounds

## Abstract

A total of 118 actinobacterial isolates were collected from the three types of termite nests (mound, carton, and subterranean nests) to evaluate their potential as a source of bioactive actinobacteria with antimicrobial activity. The highest number (67 isolates) and generic abundance (7 known genera) of actinobacterial isolates were obtained from carton nests. *Streptomyces* was the dominant genus in each type of termite nest. In the non-*Streptomyces* group, *Nocardia* was the dominant genus detected in mound and carton nests, while *Pseudonocardia* was the dominant genus in subterranean nests. A discovery trend of novel species (<99% similarity in the 16S rRNA gene sequence) was also observed in the termite nests examined. Each type of termite nest housed >20% of bioactive actinobacteria that could inhibit the growth of at least one test organism, while 12 isolates, belonging to the genera *Streptomyces*, *Amycolatopsis*, *Pseudonocardia*, *Micromonospora* and *Nocardia*, exhibited distinct antimicrobial activities. *Streptomyces* sp. CMU-NKS-3 was the most distinct bioactive isolate. It was closely related to *S. padanus* MITKK-103^T^, which was confirmed by 99% similarities in their 16S rRNA gene sequences. The highest level of extracellular antimicrobial substances was produced by the isolate CMU-NKS-3, which was grown in potato dextrose broth and exhibited a wide range (6.10×10^−4^–1.25 mg mL^−1^) of minimum inhibitory concentrations against diverse pathogens. We concluded that termite nests are an abundant source of bioactive strains of cultivable actinobacteria for future biotechnological needs.

Actinobacteria are Gram positive bacteria that possess a high guanine plus cytosine (G+C) content in their genomic DNA. They have been detected in diverse ecological niches. Their member species are known to be a main source of various bioactive compounds, accounting for 70% of all currently discovered antibiotics (7, 23). Among the antibiotics derived from actinobacteria, 75% are produced by *Streptomyces* spp. and 25% are produced by the non-*Streptomyces* group (known also as rare actinomycetes) (4). These antibiotics are produced industrially and mainly supplied for medical, pharmaceutical, and agricultural needs. On the other hand, the release of bioactive compounds to the environment through anthropogenic activity and horizontal gene transfer has led to the evolution of natural pathogens and increased drug resistance. Therefore, novel and potent bioactive compounds from novel microbial resources may overcome this pathogenic evolution. The discovery of actinobacteria from diverse and unexplored sources has also been linked to increased opportunities to obtain novel bioactive compounds (15, 34). We here aimed to explore the diversity of actinobacteria in termite nests and their potential as a source of bioactive compounds.

Termites are a group of social insects that are classified as the order Isoptera, which is closely related to cockroaches (5). They live in large colonies that comprise a king, queen, and workers, each of which have different behaviors and functions (19, 30, 38). Soil termites play an important role in circulating nutrients from decaying dead plant materials in the soil ecosystem (20, 29). Termite colonies are often found in a protective structure called a “termite nest”. These nests may be formed by diverse materials found close to their habitats. Mounds are a common type of termite nest, which are mainly constructed by the deposition of clay particles, organic carbon, and the saliva or secretions of the termites themselves. Other less common types, such as carton and subterranean nests, are also nest structures. Carton nests are mainly made from plant materials typically found on trees or in rich plant litter areas. The subterranean type is commonly found underground at an approximate depth of 50–100 cm. The various termite nests are shown in Fig. 1.

Recent studies reported no significant overlap in the gut microbial biota of soil-feeding termites and their nests or soil surrounding their nests (10, 11). Previous studies examined the microbial biota within the termite gut, and actinobacteria were identified as one of the dominant bacteria in this symbiotic lifestyle (3, 6, 20, 21, 26, 27). These symbiotic actinobacteria provide assisting functions for termites, such as nutrient cycling and exchange, and also protect termites from invading pathogens. Some of these termite-associated actinobacteria may also exhibit lignin-cellulolytic activity (3, 26, 27) and antagonistic activity against diverse pathogens (20, 21). Furthermore, some novel and prospective novel species of actinobacteria associated with termites have been reported previously (25, 32, 33). In the present study, we aimed to evaluate the generic and functional diversities of cultivable actinobacteria obtained from different types of termite nests. The biotechnological potential of the antimicrobial activity of actinobacteria was also evaluated against a set of representative pathogens found in medical and agricultural fields. The implication of different termite nests as an optimal source for potent antimicrobial actinobacteria was also discussed. The influences of some nutrition factors on the productivity of antimicrobial substances by a key actinobacterium and its further development for biotechnological applications were also assessed here.

## Materials and Methods

### Collection and preparation of termite nest samples

**Fig. 1:**
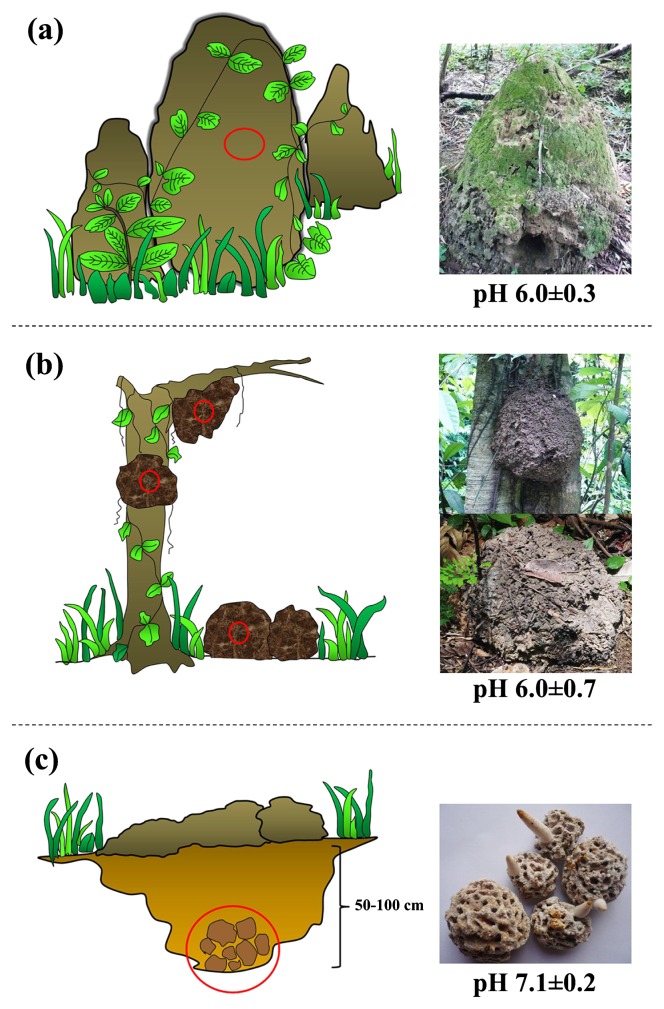
Diagram showing different types of termite nests. Three types of termite nests: mound (a), carton (b), and subterranean (c) nests, are indicated together with their average pH ± SD.

Three different termite nests, including mound, carton, and subterranean nests (Fig. 1), were collected from tropical forest and rubber tree farming areas in Phayao province, Thailand. Termite nest samples (200 g per sample from each type of termite nest) were placed in plastic bags and kept in an ice-box (for a maximum of 24 h) for transfer to the laboratory. All samples were ground into fine particles and air-dried at an ambient temperature for 7 d. Dried samples were suspended in sterilized distilled water and mixed well prior to the measurement of their pH by a pH meter (PB-10 Sartorius).

### Isolation of actinobacteria from termite nests

Dried samples (10 g each) were pretreated either by moist heating in a water bath at 50°C for 1 h (35) or by suspending with 1.5% (w/v) phenol (15). These pretreatments were performed with the aim of reducing contamination by unwanted microorganisms (low G+C and/or Gram negative bacteria). Pretreated samples were serially diluted 10-fold, and the appropriate dilutions (10^−3^ to 10^−6^) were individually spread on starch casein (SC) agar (2) or humic acid-vitamin (HV) agar (14) supplemented with nystatin, cycloheximide, and nalidixic acid at final concentrations of 100, 100, and 50 μg mL^−1^, respectively. All plates were incubated at 30°C for 4 weeks. The individual colonies that appeared were re-grown and sub-cultured on International *Streptomyces* Project Medium 2 (ISP2) agar (2) at 30°C until they were pure isolates. Pure isolates were then kept either on Hickey-Tresner agar (2) as a working stock or in 20% (v/v) glycerol at −20°C for long term storage at the Department of Biology, Faculty of Science, Chiang Mai University.

### Screening of antimicrobial actinobacteria isolated from termite nests

The antimicrobial activity of the actinobacterial isolates was evaluated by a dual culture technique against a list of test organisms indicated in Table 1. Antifungal activity against 5 phytopathogenic fungi was assessed on potato dextrose agar (PDA) (Kemma, Thailand). Briefly, an agar plug (5 mm Ø) of a test fungus grown previously on PDA at 30°C for 5 d was placed at the center of a new PDA plate, on which 2 lines (5 cm in length) of an actinobacterial isolate were individually streaked 3 cm from the fungal plug. The dual culture was incubated at 30°C for 7–14 d, and inhibition activity was observed intermittently. The plates inoculated with the fungi only served as controls. The antibacterial and anti-yeast activities of the actinobacterial isolates were assessed on nutrient agar (NA) (2) and Sabouraud agar (SBA) (2), respectively. Briefly, each actinobacterial isolate was streaked linearly at the center of each agar plate and allowed to grow at 30°C until mature colonies appeared (after approximately 3–5 d). The test organisms were streaked perpendicularly on their respective medium around a linear colony of the actinobacterial isolate. The dual culture was incubated at 37°C for the test bacteria used and at 30°C for the test yeasts used. The inhibition zone (a clear distance between the colonies of test organisms and actinobacterial isolate) that appeared was measured after 24 and 48 h of incubation. Control plates without the inoculation of actinobacteria were prepared to assess the normal growth of the test organisms on their respective agar. Distinctive antimicrobial isolates of the actinobacteria were selected based on their potential to inhibit a board range of the test organisms with relatively large inhibition zones. Of these isolates, the most distinctive antimicrobial isolate was chosen by its greater potential than that of the other isolates.

### Diversity study of actinobacteria isolated from termite nests

#### Primary identification.

The generic diversities of all actinobacteria isolated from termite nests were determined and classified using some key phenotypes from the database available in the Bergey’s Manual of Systemic Bacteriology (40). The arrangement of spores (chain or single) was examined microscopically using a cover slip-implanting technique. The cover slip was implanted on actinobacteria-growing ISP2 agar and incubated at 30°C for 7–10 d, which allowed aerial mycelia to grow on the cover slip. The cover slip was removed from the culture plate, on which the existing mycelia were then fixed by 15.5% (v/v) acetic acid for 1 min. The fixed mycelia were observed under a light microscope (Olympus SZ40, Japan) after simple staining with crystal violet. The colony morphology of the actinobacteria grown on ISP2 agar was also observed under a stereomicroscope (Olympus CH30, Japan). An analysis of 2,6-diaminopimelic acid (DAP) was carried out to determine the cell wall chemotype of the actinobacteria, following the protocol described by Ningthoujam *et al.* (24). The isomeric type of DAP together with the colony morphology were used to divide non-*Streptomyces* and unidentified genera from the *Streptomyces* group. The unidentified genera were determined according to the limited formation of aerial mycelia and spores on the agar media used. Their growth on the liquid medium used in the protocol to analyze the cell wall chemotype was also restricted.

#### Secondary identification.

Only distinctive antimicrobial isolates of actinobacteria were selected to determine their phylogenetic position. The genomic DNA of these isolates was extracted from their biomass, which was obtained after their massive growth in ISP2 broth at 30°C. The extraction was performed following the manufacturer’s instructions for the FavorPrep™ Tissue Genomic DNA Extraction Mini Sample Kit (FAVORGEN^®^, Taiwan). The concentration and purity of DNA samples were evaluated by electrophoresis on a 1% (w/v) agarose gel and visualized by staining with 0.5 μg mL^−1^ ethidium bromide. An appropriate concentration of the DNA samples was adjusted for PCR amplification of the 16S rRNA gene. *Taq* DNA polymerase and a pair of universal primers (forward primer 27 (5′-AGAGTTTGATCCTGGCTCAG-3′) and reverse primer 1,525 (5′-AAGGAGGTGWTCCARCC-3′ (R=A/G, W=A/T)) were used (18). PCR was performed according to the steps described by Adegboye and Babalola (1). PCR products were purified using the NucleoSpin^®^ Gel and PCR Clean-up Kit (MACHEREY-NAGEL, Germany) following the manufacturer’s instructions. Purified PCR products were sequenced using the facilities of First Base Laboratories, Malaysia. Almost-complete 16S rRNA gene sequences (approximately 1,500 nt) were determined and compared with the corresponding sequences available in the GenBank database (http://www.ncbi-nlm-nih.gov/) using the BLAST program. A multiple sequence alignment was carried out before the construction of a phylogenetic tree by MEGA program version 5 (36) with the neighbour-joining method (31). In pairwise comparisons, sequence similarities were computed using PHYDIT program version 1.0 (http://plaza.snu.ac.kr/jchun/phydit/).

### Effects of different fermentation media on the production of antimicrobial compounds

The most distinctive antimicrobial isolate was chosen and evaluated for its potential to form antimicrobial substances. Six different fermentation broths including potato dextrose broth (PDB) (Kemmar, Thailand), YpSs (2), AMHU-5 (22), Emerson’s Broth (EM) (2), Bennett’s Broth (BN) (2), and F-4 (8) were used to grow the isolates. Seventy milliliters of each broth was used at 30°C and shaking at 125 rpm, for 7 d. The biomass was then removed by centrifugation at 6,000 rev min^−1^ for 15 min. The supernatant of each culture broth was extracted by ethyl acetate at a ratio of 1:1 (v/v). The mixture was mixed vigorously for 5 min and left overnight, and the extraction was repeated 3 times. The ethyl acetate residual was removed by evaporation at 40°C using a rotary vacuum evaporator (Rotavapors^®^ R-215 BUCHI). The residual of antimicrobial substances was re-suspended in methanol at a concentration of 50 mg mL^−1^ and used as a crude extract for the antimicrobial test using the agar well diffusion assay against the same set of test organisms listed in Table 1.

Test bacteria were grown in nutrient broth (Difco™, USA) at 37°C and shaking at 125 rpm for 1 d, while the test yeasts were grown in Sabouraud broth (24) at 30°C and shaking at 125 rpm for 16 h, before use. The cell densities of both test organisms were then adjusted relative to the 0.5 McFarland Standard, which corresponded to 10^8^ CFU mL^−1^. Spore suspensions of phytopathogenic fungi were prepared by adding 0.85% (w/v) NaCl solution and scraping the fungal mycelia previously grown on PDA for 3–5 d. The concentration of the fungal spores was adjusted by the NaCl solution to 1–3×10^6^ spores mL^−1^ and quantified by a hemocytometer (BLAUBRAND^®^, Germany). All prepared suspensions of the test organisms were swabbed on their respective agar media including NA (for the test bacteria), SBA (for the test yeasts), and PDA (for the phytopathogenic fungi), on which wells were made. Fifty μL of the crude antimicrobial extract was added onto each agar well, while the same volumes of a negative control (methanol) and two positive controls (streptomycin [Wako Pure Chemical Industries, Japan] and benomyl [Banlee^®^, Thailand]) were added individually to the other wells. The positive controls were prepared by dissolving a powder of the antibiotics with methanol at the same concentration (50 mg mL^−1^) of the crude extract before use. Inhibition activity was observed by the appearance of inhibition zones (mm Ø), the sizes of which were recorded after an incubation at 37°C for 1 d (for the test bacteria), at 30°C for 1 d (for the test yeasts), and at 30°C for 3–5 d (for the phytopathogenic fungi). All tests were performed in triplicate.

### Determination of minimal inhibitory concentrations (MICs)

The most distinctive antimicrobial isolate was grown in 500 mL of PDB at 30°C and shaking at 125 rpm for 7 d. Its cell-free culture fluid was prepared by filtration through Whatman^®^ No. 1 filter paper, and was then extracted by ethyl acetate following a previous extraction protocol. The crude extract (5 mg mL^−1^) was serially diluted 2-fold, and appropriate dilutions (2.50 to 6.10×10^−4^ mg mL^−1^) were tested for their antimicrobial activity using the paper disc diffusion assay against the same set of test organisms listed in Table 1. Fifty μL of each dilution was loaded onto a sterilized filter paper disc, while the same volumes of negative control (methanol) and positive controls (streptomycin and benomyl) were also individually loaded onto the other paper discs. The positive controls were prepared by dissolving the powder of the antibiotics with methanol at the same concentration (5 mg mL^−1^) of the crude extract, while a set of 2-fold dilutions was performed as described above before use. MICs were determined as the lowest concentration showing inhibitory activity (appearance of an inhibition zone) against the test organisms. All tests were performed in triplicate.

### Statistical analysis

Differences in means were evaluated by a one-way analysis of variance (one-way ANOVA) using Tukey’s *post hoc* at a significant level of *P*≤0.05 within SPSS version 17.0. The statistically analyzed data of *F*-distribution values were shown elsewhere in this study. The mean values with standard deviations (SDs) were computed on the basis of an at least triplicate data set.

## Results

### Termite nest actinobacteria and their generic abundance

**Fig. 2:**
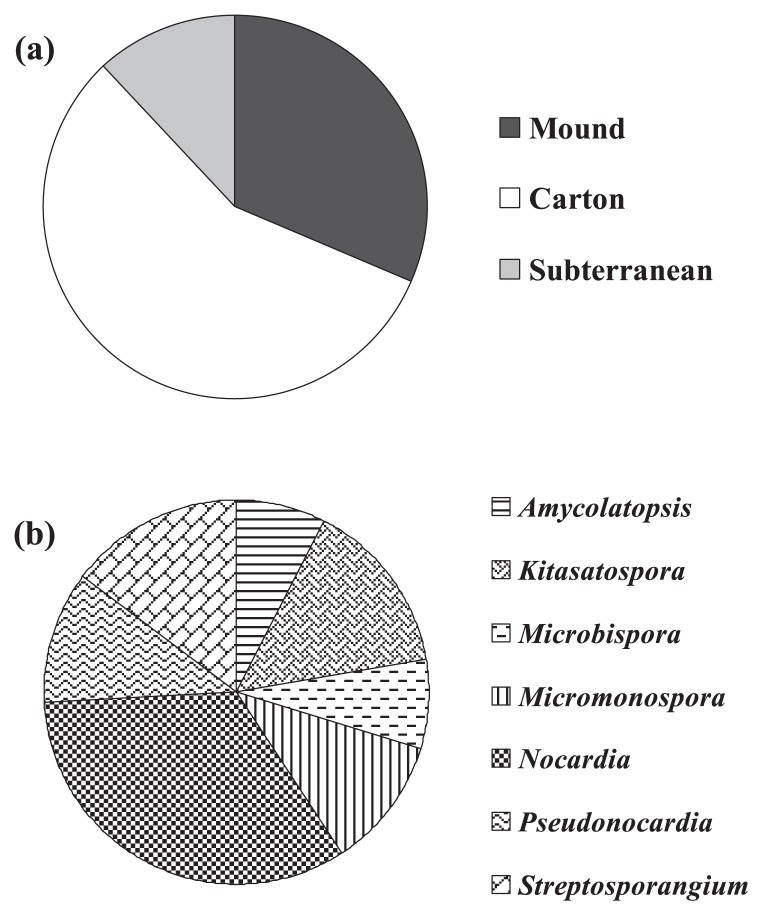
Ratio of actinobacteria derived from each type of termite nest (**a**) and primary identification of actinobacteria that belong to the non-*Streptomyces* group (**b**). This identification was performed on the basis of some key phenotypic characterizations (see also the Materials and Methods section).

**Fig. 3:**
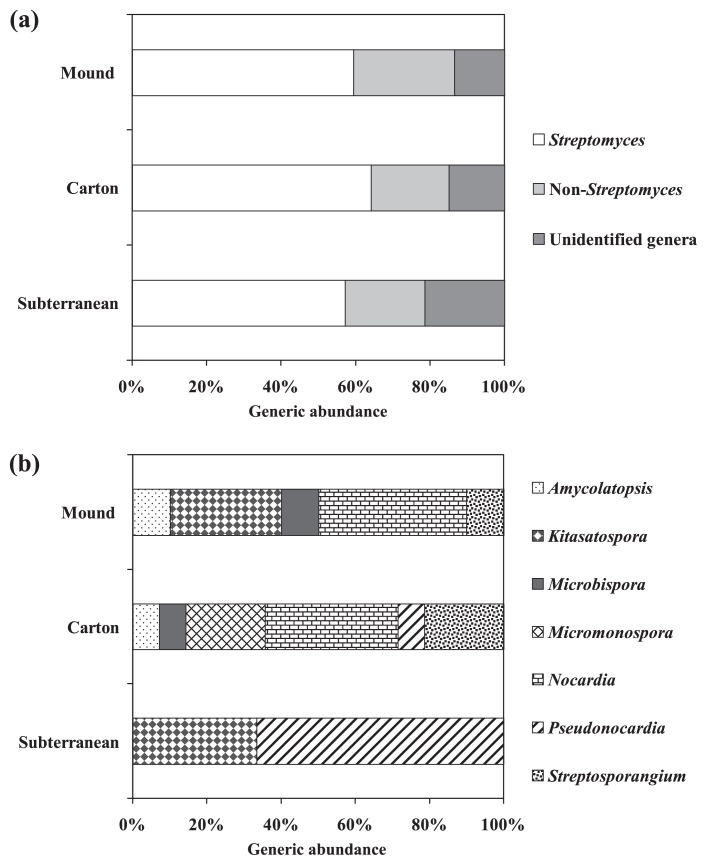
Generic diversity of actinobacteria isolated from each type of termite nest. All actinobacteria found in each type of termite nest were classified into 3 groups including *Streptomyces*, non-*Streptomyces*, and unidentified genera (**a**), while members of the non-*Streptomyces* group were primarily identified into their genera (**b**). This identification was performed on the basis of some key phenotypic characterizations (see also the Materials and Methods section).

A total of 118 actinobacterial strains were isolated from 8 termite nest samples (Table 2), while HV agar with a pretreatment of 1.5% (w/v) phenol was the optimal isolation process, giving the highest number of isolates (44.9%). A range of termite nest pH (5.3–7.2) was measured, and the average pH in each termite nest was shown in Fig. 1. Forty-five actinobacterial isolates were derived from termite nest pH 5.3–5.8, 54 isolates from pH 6.0–6.6, and 19 isolates from pH 6.6–7.2. Among all isolates, 56.8% were derived from carton nests, 31.4% were derived from mound nests, and 11.9% were derived from subterranean nests (Table 2 and Fig. 2a). All isolates within the non-*Streptomyces* group (Table 2 and Fig. 2b) were primarily identified using key phenotypic data into 7 different genera including *Amycolatopsis* (7.4%), *Kitasatospora* (14.8%), *Microbispora* (7.4%), *Micromonospora* (11.1%), *Nocardia* (33.3%), *Pseudonocardia* (11.1%) and *Streptosporangium* (14.8%). The 16S rRNA gene sequencing data of some bioactive isolates belonging to the non-*Streptomyces* group confirmed that the primary identification was accurate. For example, isolates CMU-NKS-2, CMU-NKS-51, CMU-NKS-70, and CMU-NKS-77 were primarily identified as the genera *Amycolatopsis*, *Micromonospora*, *Pseudonocardia*, and *Nocardia*, respectively. Consequently, the phylogenetic positions of these isolates were determined to be closely related to *Amycolatopsis mediterranei* NRRL B-3240^T^, *Micromonospora echinofusca* HBUM 175187^T^, *Pseudonocardia oroxyli* D10^T^, and *Nocardia harenae* WS-26^T^, respectively (Table 3). In addition, slight differences were observed in the % abundance of the actinobacterial groups (*Streptomyces*, non-*Streptomyces*, and unidentified genera) found in each type of termite nest (Table 2 and Fig. 3a). However, *Streptomyces* was the dominant genus found in every type of termite nest. The generic abundance of the non-*Streptomyces* group varied across the different types of termite nests examined, and the most diverse genera (6 genera) were detected in carton nests and the fewest (2 genera) in subterranean nests (Table 2 and Fig. 3b). Among this non-*Streptomyces* group, *Nocardia* was the dominant genus found in mound and carton nests, while *Pseudonocardia* was the dominant genus found in subterranean nests.

### Antimicrobial actinobacteria and their phylogenetic analysis

**Fig. 4:**
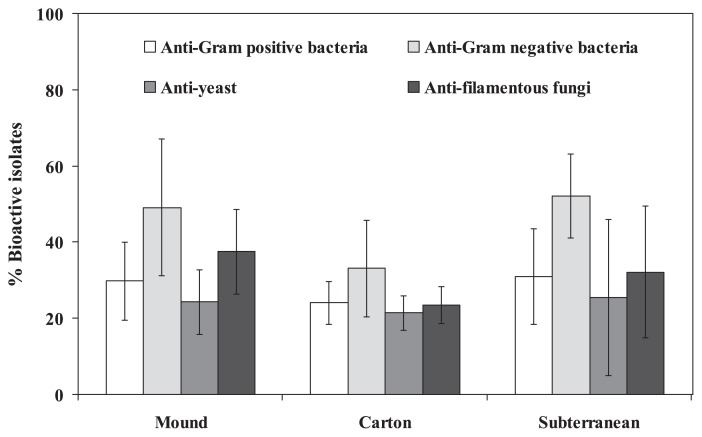
Percentage of the bioactive isolates of actinobacteria isolated from each type of termite nest. The bioactive isolates were quantified based on their capacity to inhibit the growth of at least one test organism. The graph was plotted by means ± SDs computed based on the data derived from different population numbers (numbers of termite nest samples) taken from Table 2. Statistical comparisons can be found elsewhere in this study.

**Fig. 5:**
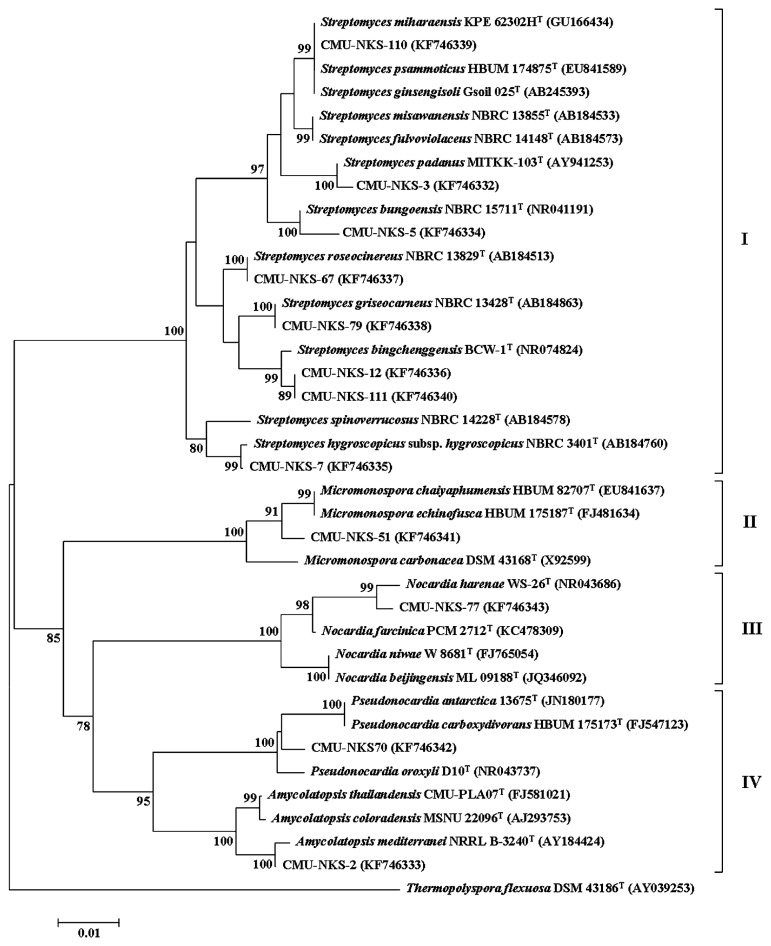
A neighbour-joining phylogenetic tree based on the 16S rRNA gene sequences, showing the relationship between the distinctive antimicrobial actinobacteria isolated from termite nests and recognized members of the class Actinobacteria. *Thermopolyspora flexuosa* DSM 43186^T^ was used as an outgroup. Bootstrap values (>50%) based on 1000 replications are shown at the branch nodes. Bar = 0.01 substitutions per nucleotide position. The accession numbers of the gene sequences are indicated in parentheses. The phylogenetic tree was categorized into 4 families: (I) *Streptomycetaceae*, (II) *Micromonosporaceae*, (III) *Nocardiaceae*, and (IV) *Pseudonocardiaceae*.

Among all the actinobacterial isolates examined, 47 isolates (39.8%) exhibited antibacterial activity, while 35 isolates (29.7%) exhibited antifungal activity. Most of these bioactive isolates were identified as *Streptomyces*, while a few isolates belonged to the non-*Streptomyces* group (Table 3). When we compared the abundance of the bioactive isolates derived from each type of termite nest, at least 20% of each could inhibit at least one test organism (Table 2 and Fig. 4), and most of the isolates could inhibit at least one Gram negative bacterium. However, no significant differences were observed in the % of bioactive isolates detected in the different types (mound, carton and subterranean) of termite nests or in the inhibition of different test organisms (Gram positive bacteria*^F1^*, Gram negative bacteria*^F2^*, yeasts*^F3^*, and filamentous fungi*^F4^*) (*F1-4*_(2,5)_ = 0.429, 1.301, 0.095, 1.261, *P*=0.001). Among all bioactive actinobacteria, the distinctive antimicrobial activities of 12 isolates were greater than those of the other isolates, as listed in Table 3. Based on the phylogenetic analysis of these distinctive isolates, the % similarities of the 16S rRNA gene sequences matched to the closest sequences available in GenBank database were 99.0% except for the isolate CMU-NKS-5, which was closely related to *Streptomyces bungoensis* NBRC 15711^T^ (98% sequence similarity, Table 3). Eight isolates belonged to the genus *Streptomyces*, while 4 isolates belonged to the non-*Streptomyces* genera, including *Amycolatopsis* (isolate CMU-NKS-2), *Micromonospora* (isolate CMU-NKS-51), *Pseudonocardia* (isolate CMU-NKS-70), and *Nocardia* (isolate CMU-NKS-77). The most distinctive antimicrobial isolate, *Streptomyces* sp. CMU-NKS-3 (which had the largest inhibition zone against most of the test organisms at the primary screening) was closely related to *Streptomyces padanus* MITKK-103^T^, as supported by 99% similarity in the 16S rRNA gene sequence. All distinctive isolates were classified into 4 families (*Streptomycetaceae*, *Micromonosporaceae*, *Nocardiaceae* and *Pseudonocardiaceae*) of the order *Actinomycetales* by phylogenetic analysis (Fig. 5), while the accession numbers (KF746332– KF746343) of their 16S rRNA gene sequences obtained after their deposition to the GenBank database can be found in Table 3.

### Influence of different nutrients on the production of antimicrobial substances by *Streptomyces* sp. CMU-NKS-3

**Fig. 6:**
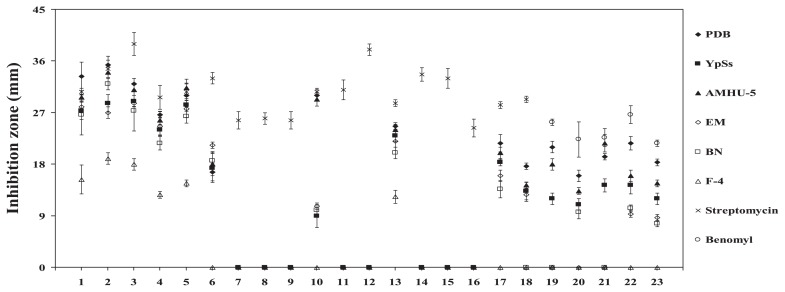
Effects of fermentation broths on the production of antimicrobial substances by *Streptomyces* sp. CMU-NKS-3. PDB, YpSs, AMHU-5, EM, BN, and F-4 were used as the fermentation broths (see also the Materials and Methods section). Streptomycin (for test bacteria and yeasts) and benomyl (for test filamentous fungi) at a final concentration of 50 mg mL^−1^ were used as the positive controls. The numbers 1–23 represent the test organisms listed in Table 1. The graph was plotted by means ± SDs computed based on the data derived from triplicate experiments. Statistical comparisons can be found elsewhere in this study.

Isolate CMU-NKS-3 showed the most distinctive antimicrobial activity against most of the test organisms examined, and was assessed for its potential to form antimicrobial substances using 6 different fermentation broths (Fig. 6). Among all the broths tested, PDB and AMHU-5 were the optimal fermentation broths for the production of antimicrobial substances by the isolate, giving significant inhibitory activity that was equal to streptomycin or benomyl against *Bacillus cereus^F5^*, *Enterococcus faecalis* ATCC 29217*^F6^*, *Staphylococcus aureus* ATCC 29213*^F7^*, MRSA*^F8^*, *Proteus mirabilis^F9^*, and *Fusarium solani^F10^* (*F5-10*_(6,14)_ = 23.828, 64.253, 51.056, 49.000, 415.597, 383.140, *P*=0.01). F-4 was the poorest fermentation broth for the production of antimicrobial substances by the isolate. Although AMHU-5 was one of the optimal fermentation broths, the crude extract derived from this broth showed significantly weaker antifungal activities against *Cryptococcus neoformans^F11^*, *Aspergillus flavus^F12^*, *Rhizoctonia solani* AG-2*^F1^*, and *Sclerotium solani^F14^* than those derived from PDB (*F11-14*_(5,12)_ = 245.169, 570.600, 181.413, 311.886, *P*=0.01). Several Gram negative bacteria were able to resist the inhibitory activity of the crude extract derived from the isolate grown in any fermentation broth (Table 1 and Fig. 6). However, the isolate showed board ranges of antimicrobial activity against the test organisms (Gram positive and Gram negative bacteria, yeasts, and filamentous fungi). The MIC values (Table 1) of the crude extract derived from the isolate grown in PDB ranged from 6.10×10^−4^ to 1.25 mg mL^−1^, while the most susceptible test organisms were both yeasts (*Candida albicans* and *Cryptococcus neoformans*). However, most Gram negative bacteria and the Gram positive bacteria, methicillin-resistant *Staphylococcus aureus* (MRSA) were the strongest antagonists among all the test organisms; no inhibition activity was observed when any concentration of the crude extract was tested. The strongest antagonist among all phytopathogenic fungi was *Aspergillus flavus*, with a MIC value of 1.25 mg mL^−1^.

## Discussion

In the present study, termite nests were shown to be an abundant source of actinobacteria; 118 actinobacterial isolates could be obtained. These actinobacteria were classified into 8 known genera in 4 families of the order *Actinomycetales*, excluding some unidentified genera. The highest number (67 isolates) and generic abundance (7 known genera) among all actinobacterial isolates were detected in carton nests. A previous study revealed that actinobacteria were microbiota in mound termite nests constructed by *Cubitermes niokoloensis* in eastern Casamance (Senegal) (11). The optimal isolation process used for actinobacteria in termite nest samples was HV agar together with a pretreatment by phenol solution. HV agar supplemented with some antibiotics was previously reported to be an optimal selective medium for the isolation of actinobacteria from diverse soil habitats (9, 14, 16, 18). In addition, the pretreatment of samples prior to the isolation of actinobacteria is an important step that prevents contamination by low G+C and/or susceptible microorganisms that live in the same samples (13, 18). Termite nests constituted by different natural materials and different termite species lead to differences in the structural and physicochemical properties of the nests. This nest construction could be a selective ecological niche for actinobacteria. Our results showed that the pH of termite nests was slightly acid to neutral, while a higher abundance of actinobacteria was found in slightly acid termite nests than in neutral ones. We hypothesized that the location of termite nests may also influence the abundance of actinobacteria; a lower number of actinobacterial isolates was obtained from subterranean termite nests (found at a depth of 50–100 cm from the ground). This may be related to the oxygen limitation within this habitat because actinobacteria are aerobic microbes. In the present study, *Streptomyces* was the dominant genus found in every type of termite nest. However, a high generic abundance (8 genera) was also observed for the non-*Streptomyces* group, while the dominant genus within this group differed between the three termite nest types examined. Based on the results of our culture-dependent study, *Nocardia* (within the family *Nocardiaceae*) was the dominant genus found in carton and mound termite nests, which was similar to a metagenomic study of microbial biota living in mound termite nests reported by Fall *et al.* (11).

Previous studies demonstrated the biotechnological potentials of microbiota associated with termites. These microbes possess diverse bioactive functions to biosynthesize several applicable metabolites such as enzymes (3, 19, 26, 27, 29, 32) and antimicrobial substances (20, 21). Few studies have evaluated the antimicrobial function of cultivable actinobacteria isolated from termite-related sources especially in the termite gut (20, 21). We here demonstrated that termite nests housed abundant antimicrobial actinobacteria. *Streptomyces* has mainly been identified as the antagonist within termite-related habitats (20, 21), which was consistent with the results obtained in the present study. However, a high variety (4 genera within 3 families) of non-*Streptomyces* genera was also detected among the antimicrobial actinobacteria derived from the 3 termite nest types in this study. The ability to obtain novel actinobacteria that also exhibited antimicrobial activity was demonstrated. A previous study identified *Saccharopolyspora pathumthaniensis* sp. nov., a novel non-*Streptomyces* actinobacterium, in the termite gut. This novel species was closely related to *Saccharopolyspora endophytica*, as supported by 99.5% similarity in the 16S rRNA gene sequences, but a low DNA-DNA relatedness value of 53.3% (33). The 16S rRNA gene sequence generally needs to be examined to phylogenetically characterize microorganisms. However, the optimal range of this gene sequence similarity for determining differences in the genomic species varies depending on each phylogenetic species. The high sequence similarity (≥99%) of the gene, but low DNA-DNA relatedness (<70%) reported within the actinobacterial members is often found such as the example of the new species mentioned above. Therefore, recent studies (17, 28) assumed the novelty of actinobacterial isolates based on a lower % similarity in the gene sequences, approximately <99%, than their matched sequences available in the GenBank database. According to this predictive criterion, the actinobacterial isolate CMU-NKS-5, which showed 98% similarity in the gene sequence, could be assumed to be a novel species within the termite nest.

Different fermentation media were tested with the aim of evaluating the potential to form bioactive compounds of the most distinctive antimicrobial isolate, *Streptomyces* CMU-NKS- 3. In a recent evaluation, potato and dextrose in PDB were the optimal nitrogen and carbon sources for the production of antimicrobial agents by the isolate. Differences in carbon and nitrogen sources together with increasing temperatures are known to be an important factor for the production of bioactive compounds by the genus *Streptomyces* (12, 37). The second optimal medium (AMHU-5) for the isolate composed of 4% carbon source (glucose) and 3% nitrogen sources (soybean meal and yeast extract) was less suitable for the production of antifungal agents. This was in contrast to a combination of 5% glucose and 1% soybean meal, which was found to be an optimal medium for the production of antifungal agents by *Streptomyces chattanoogensis* (12). Therefore, not only the nutrient composition and growing conditions, but also genetic variations in their different genomic species influence the production of bioactive compounds by actinobacteria. Isolate CMU-NKS-3 was identified as *Streptomyces padanus* MITKK-103^T^, as supported by 99% sequence similarity in the 16S rRNA gene. Previous studies reported the potential to synthesize diverse antibiotics by *S. padanus* (39, 41). Recent studies by Wang *et al.* (39) and Ziong *et al.* (41) detected antifungal agents intracellularly within the mycelia of this *Streptomyces* species, while no antifungal activity was observed with the cell-free culture fluid. This was in contrast to our results in which antifungal activity was detected in the crude extract derived from the cell-free culture broth, which had relatively low MIC values against both single cell yeasts and phytopathogenic fungi (Table 1). This suggested that either the extracellular antifungal agents we gained may differ from the previously published compounds or isolate CMU-NKS-3 may be a novel species of the genus *Streptomyces*.

## Conclusion

Termite nests represent an abundant source of cultivable actinobacteria that contribute diverse bioactive compounds for further applicable uses in the medical, pharmaceutical, and agricultural fields.

## Figures and Tables

**Table 1:** MIC values of a crude antimicrobial extract derived from *Streptomyces* sp. CMU-NKS-3.

Test organism	MIC (mg mL^−1^)		

	Crude extract from *Streptomyces* sp. CMU-NKS-3	Positive control	

		Streptomycin	Benomyl
Gram positive bacteria^a^			
*Bacillus cereus*^1^	1.22×10^−3^	0.02	ND
*Enterococcus faecalis* ATCC 29217^2^	0. 31	0.04	ND
*Micrococcus luteus*^3^	1.22×10^−3^	2.44×10^−3^	ND
*Staphylococcus aureus* ATCC 29213^4^	2.44×10^−3^	4.88×10^−3^	ND
Methicillin-resistant *Staphylococcus aureus* (MRSA)^5^	—	0.62	ND
Gram negative bacteria^b^			
*Enterobacter aerogenes*^6^	0.16	0.04	ND
*Escherichia coli* ATCC 35218^7^	—	0.08	ND
*Escherichia coli* O157:H7^8^	—	0.16	ND
*Klebsiella pneumoniae* (ESBL+)^9^	—	0.04	ND
*Proteus mirabilis*^10^	0.02	0.08	ND
*Proteus vulgaris*^11^	—	0.02	ND
*Pseudomonas aeruginosa* ATCC 27859^12^	—	0.08	ND
*Pseudomonas fluorescens*^13^	0.04	2.50	ND
*Salmonella* sp. Group D^14^	—	9.76×10^−3^	ND
*Salmonella typhae*^15^	—	0.62	ND
*Serratia marcescens*^16^	—	0.16	ND
Yeasts^c^			
*Candida albicans*^17^	6.10×10^−4^	0.31	ND
*Cryptococcus neoformans*^18^	6.10×10^−4^	0.08	ND
Filamentous fungi^d^			
*Aspergillus flavus*^19^	1.25	ND	0.31
*Colletotrichum musae*^20^	0.62	ND	0.08
*Fusarium solani*^21^	0.31	ND	0.08
*Rhizoctonia solani* AG-2^22^	0.16	ND	0.02
*Sclerotium solani*^23^	0.16	ND	0.04

a,b,c The initial stocks were kindly provided by the Central and Diagnostic Laboratory, Maharaj Nakorn Chiang Mai Hospital, Faculty of Medicine, Chiang Mai University, Thailand.

d The filamentous fungi (phytopathogens) were taken from a culture collection at the Sustainable Development of Biological Resources Lab (SDBR), Department of Biology, Faculty of Science, Chiang Mai University.

1–23 Index numbers refer to the test organisms shown in Fig. 6. Not determined (ND) and no inhibition activity (—) were also observed, while methanol was used as a negative control for all tests. Initial concentrations of the crude extract and positive controls together with their dilution factors can be found in the Materials and Methods section. Tests were performed in triplicate, and no significant difference was observed in the results derived from triplicate experiments.

**Table 2:** Actinobacteria isolated from termite nests

Termite nests	*Streptomyces*	Non-Streptomyces*							Unidentified genera	Total	Number of bioactive isolates that antagonized at least one test organism†			
	
		a	b	c	d	e	f	g			h	i	j	k
Mound (3)	22	1	3	1	0	4	0	1	5	37	3	6	2	3
											5	8	4	5
											3	4	3	6

Carton (3)	43	1	0	1	3	5	1	3	10	67	9	11	8	7
											3	7	3	3
											5	4	4	6

Subterranean (2)	8	0	1	0	0	0	2	0	3	14	2	4	1	4
											2	3	2	1

Total	73	2	4	2	3	9	3	4	18	118	32	47	27	35

The numbers in parentheses refer to the number of termite nest samples.

* The non-*Streptomyces* group comprised (a) *Amycolatopsis*, (b) *Kitasatospora*, (c) *Microbispora*, (d) *Micromonospora*, (e) *Nocardia*, (f) *Pseudonocardia*, and (g) *Streptosporangium*.

† Test organisms comprised (h) Gram positive bacteria, (i) Gram negative bacteria, (j) yeasts, and (k) filamentous fungi.

**Table 3:** Phylogenetic analysis based on the 16S rRNA gene sequences of distinctive antimicrobial actinobacteria isolated from different types of termite nests

Isolate number	Termite nests	Sequence length (nt)	Closest related bacteria in the GenBank database	Accession number	% Sequence similarity
CMU-NKS-2	Carton	1,239	*Amycolatopsis mediterranei* NRRL B-3240^T^	KF746333	99
CMU-NKS-3	Carton	1,375	*Streptomyces padanus* MITKK-103^T^	KF746332	99
CMU-NKS-5	Subterranean	1,435	*Streptomyces bungoensis* NBRC 15711^T^	KF746334	98
CMU-NKS-7	Carton	1,340	*Streptomyces hygroscopicus* subsp. *hygroscopicus* NBRC 340^T^	KF746335	99
CMU-NKS-12	Mound	1,359	*Streptomyces bingchengensis* HBUM 174849^T^	KF746336	99
CMU-NKS-51	Carton	1,345	*Micromonospora echinofusca* HBUM 175187^T^	KF746341	99
CMU-NKS-67	Mound	1,175	*Streptomyces roseocinereus* NBRC 13829^T^	KF746337	99
CMU-NKS-70	Subterranean	1,342	*Pseudonocardia oroxyli* D10^T^	KF746342	99
CMU-NKS-77	Carton	1,356	*Nocardia harenae* WS-26^T^	KF746343	99
CMU-NKS-79	Mound	1,328	*Streptomyces griseocarneus* NBRC 13428^T^	KF746338	99
CMU-NKS-110	Mound	1,071	*Streptomyces ginsengisoli* Gsoil 025^T^	KF746339	99
CMU-NKS-111	Subterranean	1,330	*Streptomyces bingchengensis* HBUM174849^T^	KF746340	99
